# Surface microhardness of three Bulk Fill resin composites previously exposed to five carbonated beverages at different times: an in vitro study

**DOI:** 10.1186/s12903-025-07055-2

**Published:** 2025-10-17

**Authors:** Luisa Estrada-Villarroel, Leonor Castro-Ramirez, María Alvino-Vales, Rita Tolmos-Valdivia, Enrique Yarasca-Berrocal, José Huamani-Echaccaya, César Cayo-Rojas

**Affiliations:** School of Stomatology, Universidad Privada San Juan Bautista, Lima and Ica, Peru

**Keywords:** Surface properties, Hardness tests, Composite resins, Dental materials, Carbonated beverages

## Abstract

**Background:**

Surface microhardness (SM) is a desirable property of any restorative material because it allows it to resist masticatory forces and any chemical challenges encountered in the oral environment. Therefore, the aim of this study was to evaluate the in vitro surface microhardness of three Bulk Fill resins previously exposed to five types of carbonated beverages at different times.

**Methods:**

This in vitro experimental study consisted of resin composite discs (*n* = 180), distributed in three groups: Tetric N-Ceram Bulk-fill, Opus Bulk Fill APS and Filtek Bulk Fill, each immersed in distilled water (control), Fanta^®^, Sprite^®^, Coca-Cola^®^, Inca-Kola^®^ and Pepsi^®^. Surface microhardness was measured before and after immersion in beverages for 1 day and 7 days. Data were subjected to statistical analysis using Games Howell’s robust ANOVA with post hoc and Friedman’s test with Bonferroni’s post hoc. Statistical significance was set at *p* < 0.05.

**Results:**

After 1 day of immersion, the Tetric N-Ceram Bulk Fill resin significantly decreased its surface microhardness after immersion in Fanta, Sprite, Coca-Cola, Inca-Kola and Pepsi (*p* < 0.05), while the Filtek Bulk Fill resin significantly decreased its SM after immersion in Pepsi and Sprite (*p* < 0.05); however, the Opus Bulk Fill resin showed no significant SM differences (*p* = 0.141). On the other hand, after 7 days of immersion, the three Bulk Fill resins significantly decreased their SM, compared to the control group (*p* < 0.001), after being immersed in the five carbonated beverages. Finally, distilled water did not significantly affect the surface microhardness of the three Bulk Fill resins over time (*p* < 0.05).

**Conclusion:**

This study highlights the susceptibility of Bulk Fill resin composites to acidic degradation caused by carbonated beverages. While short-term exposure resulted in variable effects depending on the resin type, prolonged immersion consistently led to a reduction in surface microhardness across all materials tested. These findings suggest that the chemical composition of each resin influences its resistance to erosive challenges. Although this is an in vitro study, the results may provide a basis for future investigations into how acidic diets could influence the long-term performance of Bulk Fill composites in the oral environment.

## Background

Resin composites are employed in the field of dentistry primarily due to their aesthetic appeal, resemblance to natural tooth structure, favourable mechanical properties and satisfactory clinical performance [1, 2]. Since their introduction to the market, significant efforts have been made to enhance their clinical performance, adhesion and wear resistance. However, this has proved challenging due to polymerization shrinkage, which can lead to a range of complications including fracture, secondary caries, marginal staining and postoperative sensitivity [2, 3]. The objective was to reduce the polymerization shrinkage stress of conventional resin composite and decrease the clinical time without compromising its physical properties or the quality of the restored teeth. Bulk-Fill resin composites were developed to achieve this [3, 4]. The aforementioned resins can be applied in 4–5 mm blocks, which allows a cavity to be restored in a single step. This has the advantage of reducing the number of clinical steps and the shrinkage effect. This has been achieved through modifications to the filler content and the incorporation of alternative photoinitiators [5–7].

As Bulk Fill resins have gained popularity and become more widely commercialized as a posterior restorative material, there is a need to evaluate their performance [3, 8]. Notwithstanding the aforementioned improvements, these materials remain susceptible to moisture, dietary habits and critical changes due to the consumption of acidic beverages in the oral environment [8, 9].

Carbonated beverages contain dissolved carbon dioxide, added acidity regulators (phosphoric acid, malic acid, citric acid, among others), sweeteners and natural or artificial flavorings [10, 11]. The consumption of these beverages has increased markedly in recent decades among the general population [12]. The frequency and quantity of consumption of these beverages can result in adverse effects on oral health and general wellbeing [10, 12, 13]. Consequently, restorative materials are vulnerable to the potential deterioration of their intrinsic physicochemical properties, which may be initiated by the interaction with the organic constituents of the resin matrix [8, 9, 14].

The longevity of dental restorations is dependent on the durability of the material and its physical properties, including wear resistance, adhesive strength, surface roughness (Ra), and microhardness [15]. Nevertheless, the most desirable attribute of any restorative material is its resistance to occlusal forces and chemical challenges encountered in the oral environment. This can be quantified by measuring surface microhardness [1, 5, 10].

Given that resin-based materials are susceptible to chemical agents commonly present in carbonated beverages, both short- and long-term exposure can affect their polymeric network, leading to physical and chemical degradation [16, 17]. Several studies [11, 16, 17] have demonstrated that beverages such as Coca-Cola can significantly reduce the surface microhardness of restorative materials, including glass ionomer cements and resin composites. However, the current market—both local and global—offers a broad range of carbonated drinks with varying chemical compositions and pH levels. This diversity highlights the need to evaluate the effects of other commercially available beverages to better understand their potential impact on dental restorative materials.

The aim of this study was to evaluate the in vitro surface microhardness of the bulk-fill resins Tetric N-Ceram Bulk Fill, Opus Bulk Fill APS, and Filtek Bulk Fill after exposure to the carbonated beverages Fanta, Sprite, Coca-Cola, Inca Kola, and Pepsi at different time intervals. The null hypothesis was that there would be no significant differences in the in vitro surface microhardness of the three bulk-fill resins following exposure to the five types of carbonated beverages over time.

## Methods

### Study design

Between the months of June and August 2022, this in vitro research that was both experimental and longitudinal was carried out at the School of Stomatology of the Universidad Privada San Juan Bautista in Lima, Peru, as well as at the Certified High Technology Laboratory (ISO/IEC Standard: 17025). The Institutional Research Ethics Committee (CIEI) of the Universidad Privada San Juan Bautista gave its permission to the research, which was given the number 104–2022-CIEI-UPSJB. This research was carried out in a manner that was consistent with the CRIS Guidelines, which are a checklist for reporting in-vitro studies [18].

### Sample calculation and selection

**Fig. 1:**
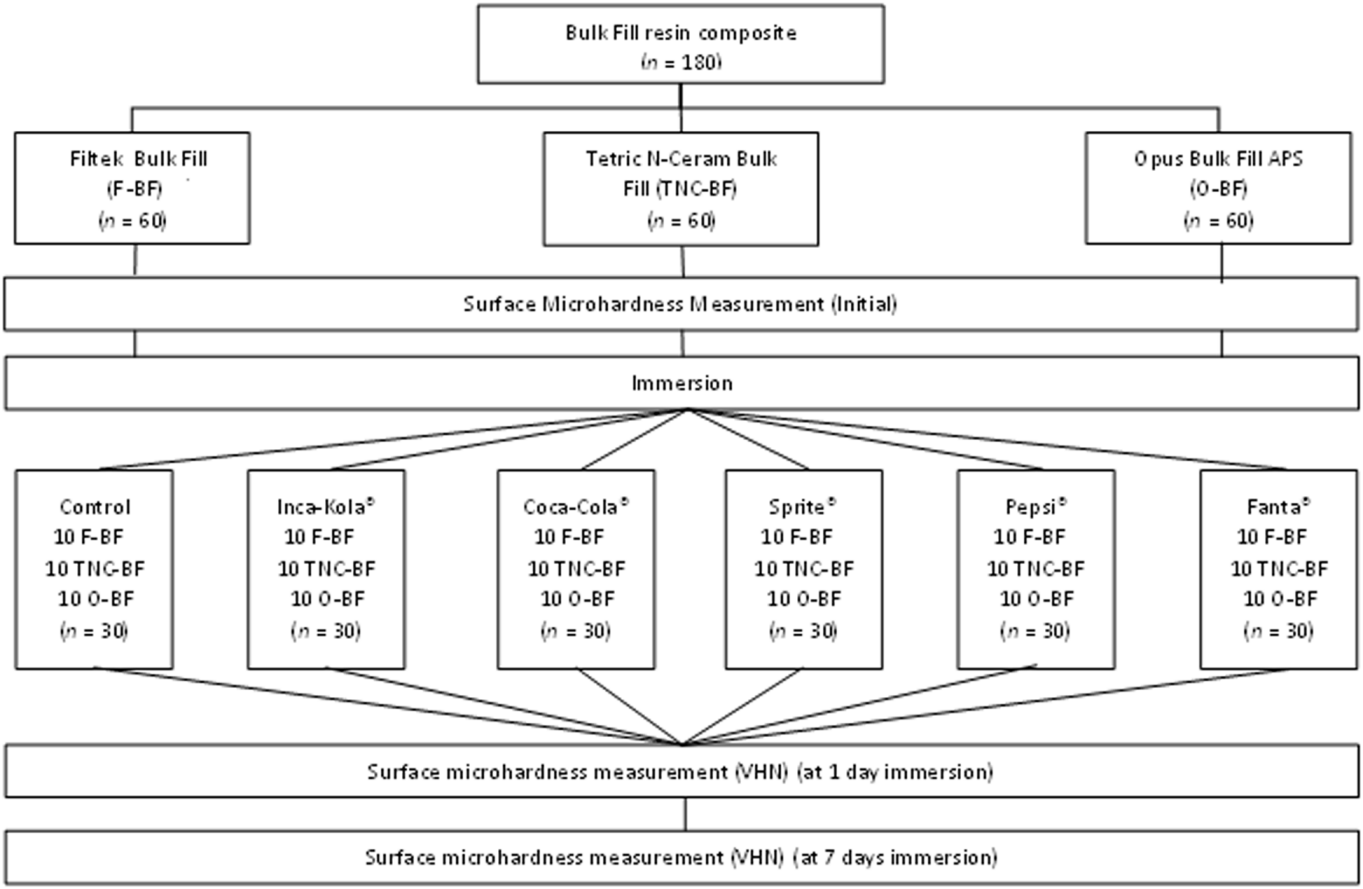
Random distribution of groups according to type of Bulk Fill resin composite and carbonated beverages

Data collected in a previous pilot investigation with five samples for each of the six subgroups of each of the three resin composites were used to calculate the total sample size, which was determined to be about 180 resin composite discs. Using the statistical programmed G*Power version 3.1.9.7, the formula for the analysis of variance was used. The significance level (α) was set at 0.05, and the statistical power (1-β) at 0.80. In addition, the effect size was set at 3.58 and there were three paired measures. Tetric N-Ceram Bulk-fill IVA (Ivoclar Vivadent, Schaan, Liechtenstein), Opus Bulk Fill APS A2 (FGM, Santa Catarina, Brazil) and Filtek Bulk Fill A2 (3 M ESPE, St. Paul, Minnesota, USA) were the three groups that evenly dispersed the 180 standardized and made-up resin discs. According to the immersion beverage each group was subjected to, they were randomly divided into six subgroups with a total of ten resin composite discs. These drinks included Pepsi^®^, Fanta^®^, Sprite^®^, Coca-Cola^®^, Inca-Kola^®^ and distilled water (Fig. 1).

### Characteristics and sample Preparation

**Fig. 2:**
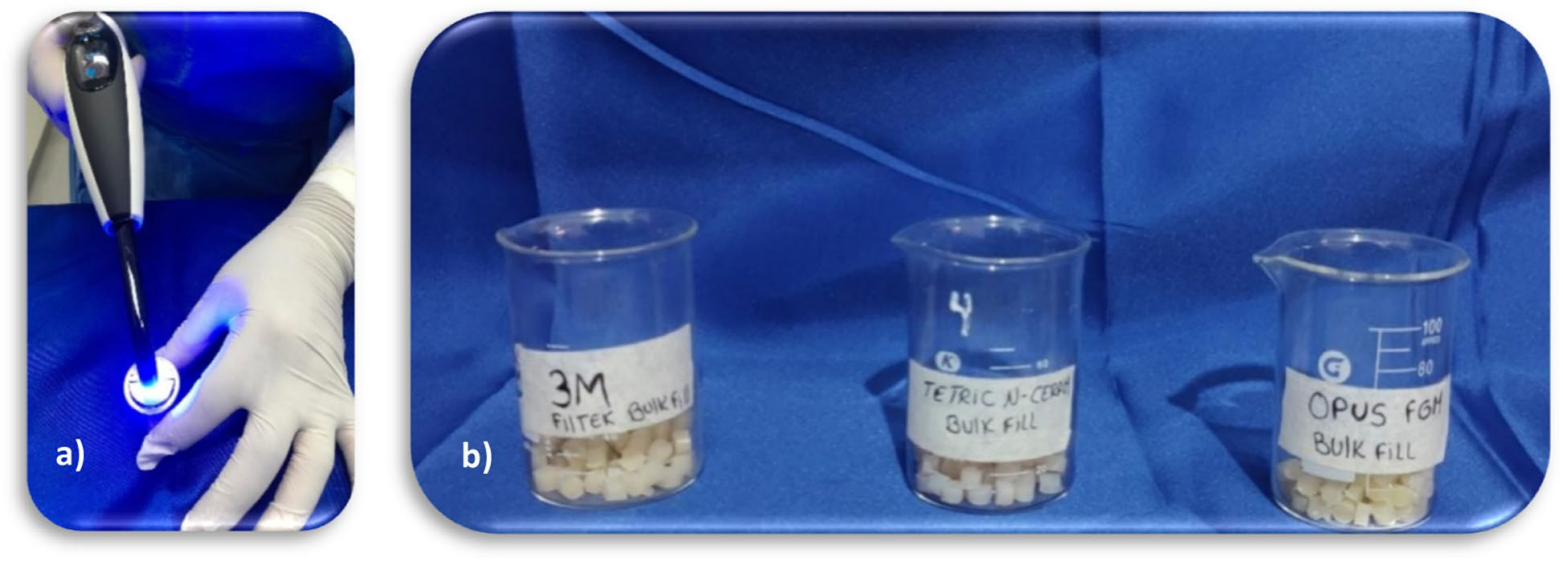
(**a**) sample processing; (**b**) samples processed by Bulk Fill resin groups

Resin composite discs were fabricated by a single operator using a metal mold with a diameter of 6 mm and a thickness of 4 mm, employing the monoblock method on a glass substrate with the assistance of a resin spatula (Hu-Fried, Chicago, Illinois, USA) [1, 6, 19, 20]. DeOx glycerin (Ultradent Inc., South Jordan, UT, USA) was then used to eliminate the oxygen-inhibited layer [1, 6, 21]. A celluloid matrix was positioned above the mold, followed by a 1 mm thick slide to guarantee parallelism of the upper and lower surfaces, which were then light-cured using a Bluephase N lamp (Ivoclar Vivadent, Schaan, Liechtenstein) at an intensity of 1200 mW/cm^2^ for 10 s [22]. The intensity was confirmed using a Bluephase Meter II radiometer (Ivoclar Vivadent, Schaan, Liechtenstein) (Fig. 2).

All Bulk Fill resin block surfaces were polished by a single operator utilizing an electric motor (EM-E6, W&H, Bürmoos, Austria), a contra-angle handpiece (NSK, Tokyo, Japan), and a four-step disc system ranging from coarse to fine grit (Sof-Lex, 3 M ESPE, St Paul, MN, USA), following the manufacturer’s guidelines [6, 21]. Subsequent to the polishing procedure, the samples were immersed in distilled water for 24 h to promote rehydration and complete the polymerization process [16, 23, 24].

All Bulk Fill resin disc surfaces were polished by a single operator utilizing an electric motor (EM-E6, W&H, Bürmoos, Austria), a contra-angle handpiece (NSK, Tokyo, Japan), and a four-step disc system ranging from coarse to fine grit (Sof-Lex, 3 M ESPE, St Paul, MN, USA), following the manufacturer’s guidelines [6, 21]. The polishing protocol was standardized by operating the coarse and medium discs at 10,000 RPM and the fine and superfine discs at 15,000 RPM. Light, consistent pressure was applied to minimize material removal. After polishing, all discs were measured again using a digital caliper, and only those with final thicknesses between 4 mm ± 0.1 mm were included in the analysis. Samples falling outside this range were discarded and replaced. Subsequent to the polishing procedure, the samples were immersed in distilled water for 24 h to promote rehydration and complete the polymerization process [16, 23, 24].

### Immersion protocol

After obtaining baseline surface microhardness measurements, the samples of each resin composite (*n* = 60) were randomly divided into six subgroups (*n* = 10): Fanta^®^, Sprite^®^, Coca-Cola^®^, Inca-Kola^®^, Pepsi^®^, and distilled water (control group) (Table 1). The carbonated beverages were used without any prior preparation, and only bottles that were within their expiration date and visibly well sealed were used [25]. Each sample was immersed in 50 mL of the corresponding beverage in individual hermetically sealed containers and stored at 37 °C for either 1 or 7 days [17, 26, 27]. All containers were kept in the dark. To prevent chemical alterations due to carbonation loss or contamination, the beverages were replaced every 24 h with freshly opened bottles [11].

**Table 1:** Compositions of the carbonated beverages used

Product	Composition	pH	Manufacturer
Pepsi^®^	Carbonated Water, Sugar, Color (Caramel E150d), Acid (Phosphoric Acid), Flavorings (including Caffeine), Sweeteners (Acesulfame K, Sucralose).	2.53	PepsiCo Inc
Sprite^®^	Carbonated Water, Sugar, Acids (Citric Acid, Tartaric Acid), Acidity Regulator (Sodium Citrates), Sweeteners (Acesulfame-K, Aspartame), Natural Lemon and Lime Flavorings.	2.64	The Coca-Cola Company
Coca-Cola^®^	Carbonated water, caramel color, flavorings, sweeteners: aspartame (0.024%) - acesulfame k (0.016%), acidulants: citric acid - phosphoric acid, preservative: sodium benzoate. Contains caffeine	2.70	The Coca-Cola Company
Fanta^®^	Carbonated Water, Orange Fruit from Concentrate (4%), Citric Acid, Sweeteners (Acesulfame K, Aspartame), Preservative (Potassium Sorbate), Malic Acid, Natural Orange Flavoring with Other Natural Flavorings, Vegetable Concentrates (Carrot, Pumpkin), Antioxidant (Ascorbic Acid), Stabilizer (Guar Gum).	2.72	The Coca-Cola Company
Inca-Kola^®^	Carbonated water, citric acid, sodium benzoate, aspartame, acesulfame potassium, caffeine, natural and artificial flavors and tartrazine.	2.90	Lindley Corporation

### Surface microhardness test

**Fig. 3:**
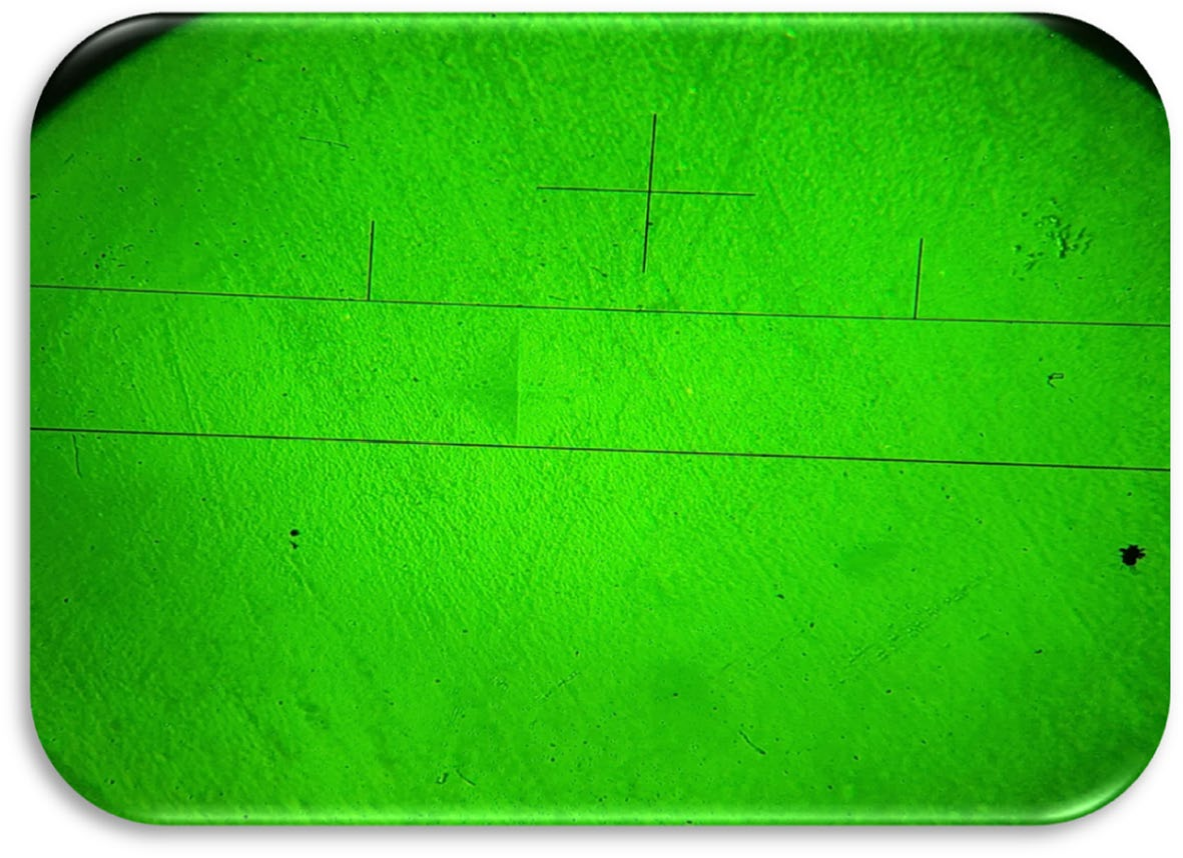
Microscopic indentation sample

The initial surface microhardness of the 180 resin composite discs was assessed before immersion in the drinks. The measurement was conducted using the Electronic Vickers microhardness tester (LG; HV-1000; Korea) with a precision of 1 micron at 40X magnification. Three indentations were created under a 100 g-f load for 10 s at equidistant sites, ensuring a minimum separation of 1 mm from the sample edges. Three consecutive measurements were recorded, and their arithmetic mean was computed. The surface microhardness value was calculated by dividing the applied force by the footprint area [kg/mm² = VH (Vickers hardness)]. Following 1 day and 7 days of immersion in the drinks, the samples were extracted, dried with absorbent paper, and the surface microhardness was re-evaluated (Fig. 3).

### Statistical analysis

The data were analyzed using the statistical program SPSS (Statistical Package for the Social Sciences Inc., IBM, NY, USA) version 28.0. The data underwent computations to determine metrics of central tendency and dispersion. The metrics comprised the mean, median, standard deviation, and interquartile range. The Shapiro-Wilk and Levene tests were used to evaluate the statistical prerequisites of normality and homogeneity of variances, respectively. The robust ANOVA with the Games-Howell post hoc analysis was used for group comparisons at each time point, whilst the Friedman test with the Bonferroni post hoc adjustment was applied for comparisons across time points. The threshold for statistical significance was established at *p* < 0.05.

## Results

Prior to immersion in Inca-Kola, Pepsi, Fanta, Sprite, Coca-Cola and distilled water, it was observed that there were no significant differences in surface microhardness in the Tetric N-Ceram Bulk Fill, Filtek Bulk Fill, and Opus Bulk Fill resin composites (*p* = 0.425, *p* = 0.775 and *p* = 0.100; respectively), thus verifying that the resin discs were standardized (Table 2).

When comparing the SM of Tetric N-Ceram Bulk Fill resin after 1 day immersion in the five different carbonated beverages, it was observed that all of them significantly decreased their SM compared to the control group (*p* < 0.001). In addition, Filtek Bulk Fill resin immersed in Pepsi and Sprite significantly reduced its SM compared to the control group (*p* < 0.05). Whereas Opus Bulk Fill resin did not decrease its SM compared to the control group (*p* = 0.141). Finally, after 7 days of immersion, all carbonated beverages significantly decreased the SM of the three bulk fill resins compared to the control group (*p* < 0.001) (Table 2).

**Table 2:** Comparison of the surface microhardness (VHN) of three resin composites immersed in different carbonated beverages according to time

Resin composite	Beverages		Baseline						At 1 day						At 7 days					
		*n*	Mean	SD	SE	95% CI		p*	Mean	SD	SE	95% CI		p*	Mean	SD	SE	95% CI		p*
						LL	UL					LL	UL					LL	UL	
TNC-BF	Inca-Kola	10	56.25	1.51	0.48	55.17	57.33	0.425	49.93^A^	1.07	0.34	49.16	50.70	< 0.001*	46.81^A^	1.95	0.62	45.41	48.21	< 0.001*
	Pepsi	10	54.70	2.24	0.71	53.10	56.30		50.79^A^	3.14	0.99	48.54	53.04		47.17^A^	4.39	1.39	44.03	50.31	
	Fanta	10	56.34	1.61	0.51	55.19	57.49		49.21^A^	4.02	1.27	46.33	52.09		44.06^A^	4.19	1.33	41.06	47.06	
	Sprite	10	56.34	1.54	0.49	55.24	57.44		51.36^A^	2.39	0.76	49.65	53.07		49.04^A^	3.53	1.11	46.52	51.56	
	Coca-Cola	10	56.05	1.74	0.55	54.80	57.30		50.96^A^	2.30	0.73	49.31	52.61		47.60^A^	2.43	0.77	45.86	49.34	
	Control	10	55.22	2.39	0.76	53.51	56.93		55.21^B^	2.36	0.74	53.52	56.90		55.27^B^	2.32	0.73	53.61	56.93	
F-BF	Inca-Kola	10	58.36	2.16	0.68	56.82	59.90	0.775	57.45^A, B^	1.11	0.35	56.66	58.24	< 0.001*	55.21^A^	2.06	0.65	53.74	56.68	< 0.001*
	Pepsi	10	59.36	1.11	0.35	58.57	60.15		56.63^A^	1.30	0.41	55.70	57.56		55.16^A^	1.79	0.57	53.88	56.44	
	Fanta	10	59.25	1.69	0.54	58.04	60.46		58.93^B^	1.76	0.56	57.67	60.19		53.73^A^	1.66	0.53	52.54	54.92	
	Sprite	10	59.48	0.85	0.27	58.87	60.09		55.81^A^	1.67	0.53	54.62	57.00		54.18^A^	2.12	0.67	52.66	55.70	
	Coca-Cola	10	59.06	1.78	0.56	57.78	60.34		57.43^A, B^	2.23	0.71	55.83	59.03		52.20^A^	2.60	0.82	50.34	54.06	
	Control	10	59.07	1.61	0.51	57.92	60.22		58.88^B^	1.25	0.39	57.99	59.77		58.56^B^	1.27	0.40	57.65	59.47	
O-BF	Inca-Kola	10	54.24	2.13	0.67	52.71	55.77	0.100	53.23	2.38	0.75	51.53	54.93	0.141	47.35^A^	3.35	1.06	44.96	49.74	< 0.001*
	Pepsi	10	55.66	1.47	0.46	54.61	56.71		54.30	2.19	0.69	52.73	55.87		46.91^A^	3.60	1.14	44.33	49.49	
	Fanta	10	54.20	1.44	0.45	53.17	55.23		52.83	1.58	0.50	51.70	53.96		46.70^A^	3.74	1.18	44.02	49.38	
	Sprite	10	54.35	1.73	0.55	53.11	55.59		52.73	1.45	0.46	51.69	53.77		49.12^A^	2.13	0.67	47.60	50.64	
	Coca-Cola	10	52.32	4.46	1.41	49.13	55.51		49.89	3.95	1.25	47.06	52.72		45.33^A^	4.26	1.35	42.28	48.38	
	Control	10	53.14	2.84	0.90	51.11	55.17		52.90	2.70	0.85	50.97	54.83		53.03^B^	3.00	0.95	50.88	55.18	

*TNC-BF* Tetric N-Ceram Bulk Fill, *F-BF* Filtek Bulk Fill, *O-BF* Opus Bulk Fill, *n* sample size, *SD* Standard Deviation, *SE* Standard Error of the mean, *95% CI* 95% Confidence Interval, *LI* Lower Limit, *UL* Upper Limit

^A^ and ^B^: different letters as superscript of the mean in each column indicated significant differences (p < 0.05) based on Games-Howell post hoc

*Based on Welch’s robust ANOVA (p < 0.05, significant differences)

Prior to immersion of the Tetric N-Ceram Bulk Fill, Filtek Bulk Fill and Opus Bulk Fill resins in distilled water, and following immersion in distilled water for 1 and 7 days, it was observed that there were no significant differences in surface microhardness. This indicates that the resin composites were not affected by immersion in distilled water over time (*p* = 0.347, *p* = 0.273 and *p* = 0.567, respectively). With regard to Tetric N-Ceram Bulk Fill, Filtek Bulk Fill and Opus Bulk Fill, it was observed that they all exhibited a significant reduction in surface microhardness over the course of seven days when immersed in Fanta, Sprite, Coca-Cola, Inca-Kola and Pepsi (*p* < 0.001) (Table 3).

**Table 3:** Variation of the surface microhardness (VHN) of three resin composites immersed in different carbonated beverages over time

Resin composite	Beverages	*n*	Baseline		At 1 day		At 7 days		p*
			Median	IQR	Median	IQR	Median	IQR	
TNC-BF	Inca-Kola	10	56.45^a^	2.35	50.25^a, b^	1.68	47.45^b^	3.60	< 0.001*
	Pepsi	10	54.70^a^	3.20	49.75^b^	4.85	46.80^b^	6.65	< 0.001*
	Fanta	10	56.95^a^	2.45	50.50^a, b^	8.23	43.80^b^	8.00	< 0.001*
	Sprite	10	56.95^a^	2.18	51.55^a, b^	4.72	49.00^b^	6.70	< 0.001*
	Coca-Cola	10	56.10^a^	2.65	51.40^a, b^	4.35	46.65^b^	3.65	< 0.001*
	Control	10	54.70	3.68	54.70	3.40	54.85	3.40	0.347
F-BF	Inca-Kola	10	58.90^a^	3.53	57.60^a, b^	2.28	55.65^b^	1.83	< 0.001*
	Pepsi	10	59.25^a^	1.88	56.80^a, b^	1.83	55.10^b^	2.95	< 0.001*
	Fanta	10	59.30^a^	2.38	58.75^a^	2.85	53.55^b^	2.90	0.001*
	Sprite	10	59.50^a^	1.30	56.15^a, b^	2.45	54.05^b^	4.28	< 0.001*
	Coca-Cola	10	59.40^a^	3.53	57.20^a, b^	3.85	51.75^b^	3.75	< 0.001*
	Control	10	59.30	2.83	58.95	1.85	58.15	2.00	0.273
O-BF	Inca-Kola	10	53.90^a^	4.53	53.40^b^	3.85	46.10^b^	5.33	< 0.001*
	Pepsi	10	55.20^a^	2.13	54.60^a, b^	4.15	46.35^b^	6.25	< 0.001*
	Fanta	10	54.00^a^	2.50	52.75^a, b^	2.55	46.95^b^	6.33	< 0.001*
	Sprite	10	54.20^a^	2.48	52.50^a, b^	2.28	49.65^b^	3.53	< 0.001*
	Coca-Cola	10	51.95^a^	7.40	49.85^a, b^	7.75	44.95^b^	8.05	< 0.001*
	Control	10	53.75	4.95	53.45	4.18	53.90	5.60	0.567

*TNC-BF* Tetric N-Ceram Bulk Fill, *F-BF* Filtek Bulk Fill, *O-BF* Opus Bulk Fill, *n* sample size, *IQR* Interquartile Range

^a^ and ^b^: different letters as superscript of the median in each row indicated significant differences (p < 0.05) based on Bonferroni post hoc

*Based on Friedman’s test (p < 0.05, significant differences)

## Discussion

Surface microhardness represents the most desirable characteristic of restorative materials, as they must withstand the forces of occlusion and any chemical challenges in the oral environment [1, 5, 10]. The increased consumption of carbonated beverages by the population may have a detrimental effect on the properties of restorative materials and dental health [10, 12]. The aim of this study was to assess the in vitro surface microhardness of three Bulk Fill resins previously exposed to five types of carbonated beverages at varying time points. The findings revealed that the Filtek Bulk Fill and Tetric N-Ceram Bulk Fill resins exhibited a notable decline in surface microhardness following 1 and 7 days of immersion in carbonated beverages, respectively. In addition, the Opus Bulk Fill demonstrated a more pronounced reduction in surface microhardness after 7 days of exposure. Consequently, the null hypothesis was rejected.

Bulk-fill resins have been developed with the objective of simplifying restorative techniques, as they can be placed in the cavity preparation and light-cured in a single 4–5 mm increment [5–7]. Consequently, manufacturers employ diverse methodologies to enhance the composition of these materials, thereby facilitating light curing across the entire increment. One such approach entails modifying the shape of the filler particles [28]. Another strategy that has been implemented is the use of a photoinitiator that serves to amplify the polymerization capacity, thereby increasing both the degree of conversion and the depth of cure, resulting in a good quality polymer [28].

The results obtained in the present study showed that, during the first day of immersion, Tetric N-Ceram Bulk Fill significantly decreased its SM with all five beverages. This may be due to the lower percentage of filler particles in its composition (76 wt%; 54 vol%), compared to Opus Bulk Fill (76.5 wt%; 58.4 vol%) and Filtek Bulk Fill (76.5 wt%; 58.5 vol%) [1, 20]. However, it is worth mentioning that Filtek Bulk Fill also significantly decreased its SM, but only with Pepsi and Sprite, which may be due to the pH of the beverages, as both had the lowest pH compared to the others. It has been documented that acidic foods and beverages with a low pH induce erosive wear and surface disintegration, which consequently affects the hardness of resins [11, 29]. Furthermore, Hwang et al. [30] have demonstrated that a reduction in pH results in a notable decline in the microhardness of resin composite [31]. Conversely, it is likely that Opus Bulk Fill was not affected, as this resin composite uses a novel advanced polymerization system (APS) technology that reduces the quantity of camphorquinone through the incorporation of alternative initiators and manufacturers’ proprietary co-initiators, which enhance the polymerization capacity and elevate both the degree of conversion and depth of cure. This would assist in enhancing its mechanical and surface properties [1, 21, 28, 32].

After 7 days of immersion, the three Bulk Fill resins significantly decreased SM with the 5 carbonated beverages, which is evident that all three resins were affected by the acidic pH of the 5 beverages, thus proving that the pH of the beverages compromises the hardness of the materials [11, 29]. Furthermore, SM values can also be compromised in the short term (1 day), depending on resin characteristics such as content, filler distribution and resin matrix composition [17]. Conversely, when the three resin composites were immersed in distilled water for one and seven days, no significant differences in SM were observed over time. This finding was inconsistent with the observations of other researchers [20, 23, 33], who have noted that, despite maintaining a neutral pH, Bis-GMA and UDMA are highly susceptible to water absorption, which can result in the softening and degradation of the matrix. Furthermore, disbonding of filler particles, release of unreacted monomers, and subsequent hydrolytic degradation may be responsible for the observed decrease in surface hardness after immersion in different solutions [11, 20, 23, 33]. This discrepancy may be attributed to the oxygen inhibition layer (OIL) inhibition employed in the present study. Previous research has demonstrated that the application of a protective layer of glycerin to the final resin composite enhances surface hardness, thereby improving the quality and longevity of resin composites [21, 34]. Furthermore, it was observed that only Pepsi significantly decreased the SM of the Tetric N-Ceram Bulk Fill resin after one day of immersion, remaining similar until day seven of immersion. This could be attributed to its buffering capacity, defined as the ability of the beverage to resist a change in pH. It has been established that the greater the buffering capacity of a beverage, the greater its erosive effect. In a study by Carvalho et al. [35], it was demonstrated that Pepsi exhibited a higher buffering capacity than Coca-Cola in both its original and light formulations.

Finally, it was observed that Inca-Kola significantly reduced the SM of the Opus Bulk Fill resin after one day of immersion, and that this reduction remained constant until the seventh day of immersion. This can be attributed to the fact that Inca-Kola is a beverage composed mainly of citric acid, while the other beverages contain different proportions of malic acid, phosphoric acid and others. In addition, the presence of different types and concentrations of acids may also help to elucidate the discrepancy in pH and buffer effect between the different beverages used [35]. It has been shown that beverages with a higher concentration of citric acid have a more pronounced erosive effect than those with a higher proportion of phosphoric acid [36, 37]. It should be noted that the microhardness of resins is influenced by a number of factors, such as the volume or weight of the inorganic particles, the constitution of the polymer matrix, the morphology, size and distribution of the filler particles and the quality of the transmitted light [20, 28]. Consequently, the effect of different beverages may vary between different resin composites.

The results obtained in this study are consistent with the findings reported by Bengal et al. [29], Borges et al. [14], and Poggio et al. [17], where SM significantly decreased for all resin composites analyzed, despite the use of different resin formulations. This consistency with previous studies can be attributed to the erosive action of acidic media present in carbonated beverages, which affect the organic matrix of composite resins. The low pH and the presence of acids such as phosphoric, citric, or carbonic acid promote surface demineralization, softening of the polymer matrix, and potential filler particle dissolution or degradation.

The findings of this study provide valuable insight into how different carbonated beverages affect the SM of bulk-fill resins, which is critical in clinical dental practice. This information may assist clinicians in making more informed decisions when selecting restorative materials for patients with dietary habits that involve frequent consumption of carbonated drinks. Furthermore, it highlights the importance of educating patients on the potential erosive effects of these beverages on dental restorations, thereby contributing to improved treatment longevity and long-term oral health.

Despite the limited availability of studies examining the specific influence of various carbonated beverage components on the properties of composite resins, the present study addresses this gap. While most previous research [11, 16, 17] has focused on comparing a single carbonated beverage—typically Coca-Cola—with non-carbonated drinks such as coffee or tea, this study broadens the scope by evaluating multiple commercially available carbonated beverages. In doing so, it contributes substantially to scientific knowledge and opens new avenues for future research on the interaction between restorative materials and commonly consumed acidic beverages.

This study included bulk-fill resin composites with different photoinitiators. Tetric N-Ceram Bulk Fill contains alternative photoinitiators designed to enhance light polymerization, such as Ivocerin (a dibenzoyl germanium derivative) and monoacylphosphine oxide (TPO). In contrast, Opus Bulk Fill APS uses a novel technology called Advanced Polymerization System (APS), which reduces the amount of camphorquinone by incorporating other types of initiators and co-initiators, whose exact composition remains proprietary [1, 6, 21]. Filtek Bulk Fill, on the other hand, relies exclusively on camphorquinone as its photoinitiator. Differences in the composition of these resins may influence the degree of monomer conversion and the depth of cure under LED irradiation, which in turn could positively affect their mechanical and surface properties [1, 6, 21]. Additionally, the selected carbonated beverages—Coca-Cola, Pepsi, Sprite, and Fanta—are among the most widely consumed by the global youth population [38, 39], while Inca-Kola was included due to its high popularity in the Peruvian market [40].

One strength of the study design is the confirmation that all resin specimens exhibited standardized SM at baseline, as evidenced by similar initial values prior to immersion in carbonated beverages. This contrasts with previous studies that did not report baseline SM measurements [17, 25, 27]. In addition, microhardness was assessed using the Vickers hardness test, a method considered more precise and sensitive than Brinell or Rockwell tests for evaluating dental materials [11]. Another notable strength is the inclusion of five distinct carbonated beverages, in contrast to prior studies that often evaluated only one drink [11, 16, 17]. Furthermore, the specimens were immersed for seven consecutive days (168 h) [41], a duration chosen to simulate long-term oral exposure. Assuming that a sip of soda results in approximately one minute of contact with the oral cavity and is consumed five times per day from the same bottle, this would total five minutes of daily exposure. Under this assumption, the seven-day immersion period can be considered roughly equivalent to five years of cumulative exposure in the oral environment [31].

One of the main limitations of this study lies in its in vitro nature, which restricts the direct extrapolation of results to real clinical conditions, where factors such as saliva, temperature, dynamic pH, and masticatory forces influence the behavior of restorative materials. In addition, the analysis was limited to five carbonated beverages, without accounting for the wide variety of commercially available products with different chemical compositions. The exposure times used do not necessarily reflect real consumption patterns, and no artificial aging methods, such as thermocycling, were applied to simulate long-term composite degradation. Furthermore, only surface microhardness was evaluated, without considering other relevant physical-mechanical properties. Lastly, the findings are limited to three specific bulk-fill resins and cannot be generalized to all available brands or formulations. These limitations should be taken into account when interpreting the results and highlight the need for complementary in vivo studies to validate and expand upon these findings.

## Conclusion

This study highlights the susceptibility of Bulk Fill resin composites to acidic degradation caused by carbonated beverages. While short-term exposure resulted in variable effects depending on the resin type, prolonged immersion consistently led to a reduction in surface microhardness across all materials tested. These findings suggest that the chemical composition of each resin influences its resistance to erosive challenges. Although this is an in vitro study, the results may provide a basis for future investigations into how acidic diets could influence the long-term performance of Bulk Fill composites in the oral environment.

## Data Availability

The datasets used and/or analysed during the current study are available from the corresponding author on reasonable request.

## Abbreviations

F-BF: Filtek Bulk Fill
IQR: Interquartile Range
O-BF: Opus Bulk Fill
SM: Surface microhardness
SPSS: Statistical Package for the Social Sciences
TNC-BF: Tetric N-Ceram Bulk Fill

## References

[1] Berto-Inga J, Santander-Rengifo F, Ladera-Castañeda M, López-Gurreonero C, Castro Pérez-Vargas A, Cornejo-Pinto A, Cervantes-Ganoza L, Cayo-Rojas C. Surface microhardness of Bulk-Fill resin composites handled with gloves. Int Dent J. 2023;73(4):489–95. 10.1016/j.identj.2022.10.005. 36404177 10.1016/j.identj.2022.10.005 PMC10350598

[2] Rodríguez HA, Kriven WM, Casanova H. Development of mechanical properties in dental resin composite: effect of filler size and filler aggregation state. Mater Sci Eng C Mater Biol Appl. 2019;101:274–82. 10.1016/j.msec.2019.03.090. 31029321 10.1016/j.msec.2019.03.090

[3] Strini BS, Marques JFL, Pereira R, Sobral-Souza DF, Pecorari VGA, Liporoni PCS, Aguiar FHB. Comparative evaluation of Bulk-Fill composite resins: Knoop Microhardness, diametral tensile strength and degree of conversion. Clin Cosmet Investig Dent. 2022;14:225–33. 10.2147/CCIDE.S376195. 35957701 10.2147/CCIDE.S376195 PMC9359371

[4] Cayo C, Llancari L, Mendoza R, Cervantes L. Marginal filling and adhesive resistance of bulk fill resin applying 18% Edta gel compared with 37% phosphoric acid gel in vitro dental conditioning. J Oral Res. 2019;8(3):228–35.

[5] Alshehri A, Alhalabi F, Robaian A, Abuelqomsan MAS, Alshabib A, Ismail E, Alzamil F, Alotaibi N, Algamaiah H. No-Cap flowable Bulk-Fill composite: Physico-Mechanical assessment. Polym (Basel). 2023;15(8):1847. 10.3390/polym15081847. 10.3390/polym15081847 PMC10144174 37111994

[6] Gaviria-Martinez A, Castro-Ramirez L, Ladera-Castañeda M, Cervantes-Ganoza L, Cachay-Criado H, Alvino-Vales M, Garcia-Luna G, López-Gurreonero C, Cornejo-Pinto A, Cayo-Rojas CF. Surface roughness and oxygen inhibited layer control in bulk-fill and conventional nanohybrid resin composites with and without polishing: in vitro study. BMC Oral Health. 2022;22(1):258. 10.1186/s12903-022-02297-w. 35754035 10.1186/s12903-022-02297-w PMC9235274

[7] Pirmoradian M, Hooshmand T, Jafari-Semnani S, Fadavi F. Degree of conversion and microhardness of bulk-fill dental composites polymerized by LED and QTH light curing units. J Oral Biosci. 2020;62(1):107–13. 10.1016/j.job.2019.12.004. 31863827 10.1016/j.job.2019.12.004

[8] Gehlot PM, Sudeep P, Manjunath V, Annapoorna BM, Prasada LK, Nandlal B. Influence of various desensitizing mouthrinses and simulated toothbrushing on surface roughness and microhardness of tetric N-Ceram Bulk-Fill resin composite: an in vitro study and scanning electron microscope analysis. Eur J Dent. 2022;16(4):820–7. 10.1055/s-0041-1739547. 35176786 10.1055/s-0041-1739547 PMC9683869

[9] Tseng CC, Lin PY, Kirankumar R, Chuang ZW, Wu IH, Hsieh S. Surface degradation effects of carbonated soft drink on a resin based dental compound. Heliyon. 2021;7(3):e06400. 10.1016/j.heliyon.2021.e06400. 33869827 10.1016/j.heliyon.2021.e06400 PMC8035514

[10] Ganesh S, Ganesh SB, Jayalakshmi S. Effect of carbonated beverages on flexural strength property of restorative glass ionomer cement. J Adv Pharm Technol Res. 2022;13(Suppl 1):S186–9. 10.4103/japtr.japtr_265_22. 36643106 10.4103/japtr.japtr_265_22 PMC9836160

[11] Barve D, Dave PN, Gulve MN, Meera Sahib MA, Naz F, Shahabe SA. Effect of commonly consumed beverages on microhardness of two types of composites. Int J Clin Pediatr Dent. 2020;13(6):663–7. 10.5005/jp-journals-10005-1854. 33976493 10.5005/jp-journals-10005-1854 PMC8060943

[12] Tahmassebi JF, BaniHani A. Impact of soft drinks to health and economy: a critical review. Eur Arch Paediatr Dent. 2020;1(1):109–17. 10.1007/s40368-019-00458-0. 10.1007/s40368-019-00458-0 31177478

[13] Sushma B, Ganesh SB, Jayalakshmi S. Effect of carbonated beverages on flexural strength of composite restorative material. J Adv Pharm Technol Res. 2022;13(Suppl 1):S160–3. 10.4103/japtr.japtr_264_22. 36643132 10.4103/japtr.japtr_264_22 PMC9836129

[14] Borges MG, Soares CJ, Maia TS, Bicalho AA, Barbosa TP, Costa HL, Menezes MS. Effect of acidic drinks on shade matching, surface topography, and mechanical properties of conventional and bulk-fill composite resins. J Prosthet Dent. 2019;121(5):e8681–868. 10.1016/j.prosdent.2019.02.006. 10.1016/j.prosdent.2019.02.006 31010591

[15] Guedes APA, Oliveira-Reis B, Catelan A, Suzuki TYU, Briso ALF, Santos PHD. Mechanical and surface properties analysis of restorative materials submitted to erosive challenges in situ. Eur J Dent. 2018;12(4):559–65. 10.4103/ejd.ejd_188_18. 30369803 10.4103/ejd.ejd_188_18 PMC6178688

[16] Moyin S, Lahiri B, Sam G, Nagdev P, Kumar NN. Evaluation of the impact of acidic drink on the microhardness of different esthetic restorative materials: an in vitro study. J Contemp Dent Pract. 2020;21(3):233–7. 10.5005/p.journals-10024-2753. 32434966

[17] Poggio C, Viola M, Mirando M, Chiesa M, Beltrami R, Colombo M. Microhardness of different esthetic restorative materials: evaluation and comparison after exposure to acidic drink. Dent Res J. 2018;15(3):166–72. 10.4103/1735-3327.231863. PMC5958532 29922334

[18] Krithikadatta J, Gopikrishna V, Datta M. CRIS guidelines (Checklist for reporting In-vitro Studies): A concept note on the need for standardized guidelines for improving quality and transparency in reporting in-vitro studies in experimental dental research. J Conserv Dent. 2014;17(4):301–4. 10.4103/0972-0707.136338. 25125839 10.4103/0972-0707.136338 PMC4127685

[19] De Arruda BM, Bassi JC, Vitti R, Scatolin R. Color stability of bulk fill composite resins submitted to coffee staining. Braz Dent Sci. 2021;24(1):1–7. 10.14295/bds.2021.v24i1.2304.

[20] Espíndola-Castro LF, Durão MA, Pereira TVG, Cordeiro AKB, Monteiro GQM. Evaluation of microhardness, sorption, solubility, and color stability of bulk fill resins: A comparative study. J Clin Exp Dent. 2020;12(11):e1033–8. 10.4317/jced.57599. 33262868 10.4317/jced.57599 PMC7680569

[21] Carrillo-Marcos A, Salazar-Correa G, Castro-Ramirez L, Ladera-Castañeda M, López-Gurreonero C, Cachay-Criado H, Aliaga-Mariñas A, Cornejo-Pinto A, Cervantes-Ganoza L, Cayo-Rojas CF. The microhardness and surface roughness assessment of Bulk-Fill resin composites treated with and without the application of an Oxygen-Inhibited layer and a Polishing system: an in vitro study. Polymers). 2022;14(15):3053. 10.3390/polym14153053. 35956567 10.3390/polym14153053 PMC9370367

[22] López-Torres J, Hernández-Caba K, Cervantes-Ganoza L, Ladera-Castañeda M, Martínez-Campos R, Solís-Dante F, Briceño-Vergel G, Cayo-Rojas C. Microleakage of class II Bulk-Fill resin composite restorations cured with Light-Emitting diode versus quartz Tungsten-Halogen light: an in vitro study in human teeth. Biomedicines. 2023;11(2):556. 10.3390/biomedicines11020556. 36831092 10.3390/biomedicines11020556 PMC9953121

[23] Şişmanoğlu S, Sengez G. Effects of acidic beverages on color stability of Bulk-Fill composites with different viscosities. Odovtos - Int J Dent. 2021;24(2):90–9. 10.15517/ijds.2022.49149.

[24] Tighiceanu C, Bulai ER, Iatcu OC, Dulucheanu C, Nemtoi A. Effect of vegetable juices on properties of two resin composites used for dental caries management. Med (Kaunas). 2023;59(4):774. 10.3390/medicina59040774. 10.3390/medicina59040774 PMC10142735 37109732

[25] Faris TM, Abdulrahim RH, Mahmood MA, Mhammed Dalloo GA, Gul SS. In vitro evaluation of dental color stability using various aesthetic restorative materials after immersion in different drinks. BMC Oral Health. 2023;23(1):49. 10.1186/s12903-023-02719-3. 36710338 10.1186/s12903-023-02719-3 PMC9884413

[26] Scribante A, Bollardi M, Chiesa M, Poggio C, Colombo M. Flexural Properties and Elastic Modulus of Different Esthetic Restorative Materials: Evaluation after Exposure to Acidic Drink. Biomed Res Int. 2019;2019:5109481. 10.1155/2019/5109481. 30863779 10.1155/2019/5109481 PMC6378791

[27] Abouelmagd DM, Basheer RR. Microhardness evaluation of microhybrid versus nanofilled resin composite after exposure to acidic drinks. J Int Soc Prevent Communit Dent. 2022;12(3):353–9. 10.4103/jispcd.JISPCD_66_22. 10.4103/jispcd.JISPCD_66_22 PMC9369782 35966915

[28] Viana MOS, Lopes TCS, Lopes MABS, Pires LGS, Campos MRO, Brandim AS. Evaluation of the microhardness of different types of bulk fill resins. EJDENT. 2023;4(5):7–11. 10.24018/ejdent.2023.4.5.284.

[29] Bengal S, Badole GP, Shenoi PR, Kubde R, Shahu S. Evaluation of surface roughness and microhardness of Bulk-fill and nanohybrid composite after exposure to different beverages at various time intervals - An in vitro study. Ann Afr Med. 2024;23(3):466–73. 10.4103/aam.aam_157_23. 39034574 10.4103/aam.aam_157_23 PMC11364335

[30] Hwang S, Chung SH, Lee JT, Kim YT, Kim YJ, Oh S, Yeo IL. Influence of Acid, Ethanol, and anthocyanin pigment on the optical and mechanical properties of a nanohybrid dental composite resin. Mater (Basel). 2018;11(7):1234. 10.3390/ma11071234. 10.3390/ma11071234 PMC6073282 30021991

[31] Szalewski L, Wójcik D, Bogucki M, Szkutnik J, Różyło-Kalinowska I. The influence of popular beverages on mechanical properties of composite resins. Materials. 2021;14(11):3097. 10.3390/ma14113097. 34198751 10.3390/ma14113097 PMC8201062

[32] FGM. Opus Bulk Fill APS | FGM. 2020. Available from: https://fgmdental.es/producto/composite-baja-tension-contraccionopus-bulk-fill-aps/. (Accessed 13 July 2022).

[33] Somayaji SK, Amalan A, Ginjupalli K. Effect of different acidic beverages on microhardness of nanohybrid composite, Giomer, and microhybrid composite. World J Dent. 2016;7(3):126–8. 10.5005/jp-journals-10015-1380.

[34] Ferreto-Gutiérrez I, Hernández-Mata A. Mathematical-Physical description of the oxygen-inhibited layer (OIL) in nanofilled dental polymers. Odovtos. 2024;26(1):65–75.

[35] De Carvalho Sales-Peres SH, Magalhães AC, de Andrade Moreira Machado MA, Buzalaf MA. Evaluation of the erosive potential of soft drinks. Eur J Dent. 2007;1(1):10–3. 19212490 PMC2612950

[36] West NX, Hughes JA, Addy M. The effect of pH on the erosion of dentine and enamel by dietary acids in vitro. J Oral Rehabil. 2001;28(9):860–4. 10.1046/j.1365-2842.2001.00778.x. 11580825 10.1046/j.1365-2842.2001.00778.x

[37] Lafuente D, Abad K. Influencia de Bebidas Gaseosas En La integridad de Márgenes En restauraciones de Resina compuesta. Odovtos - Int J Dent Sci. 2014;16:115–23.

[38] Hu H, Song J, MacGregor GA, He FJ. Consumption of soft drinks and overweight and obesity among adolescents in 107 countries and regions. JAMA Netw Open. 2023;6(7):e2325158. 10.1001/jamanetworkopen.2023.25158. 37486630 10.1001/jamanetworkopen.2023.25158 PMC10366702

[39] Yang L, Bovet P, Liu Y, Zhao M, Ma C, Liang Y, Xi B. Consumption of carbonated soft drinks among young adolescents aged 12 to 15 years in 53 low- and middle-income countries. Am J Public Health. 2017;107(7):1095–100. 10.2105/AJPH.2017.303762. 28520485 10.2105/AJPH.2017.303762 PMC5463205

[40] Alcalde MC. Between Incas and indians: Inca Kola and the construction of a Peruvian-global modernity. J Consum Cult. 2009;9(1):31–54. 10.1177/1469540508099700.

[41] von Fraunhofer JA, Rogers MM. Dissolution of dental enamel in soft drinks. Gen Dent. 2004;52(4):308–12. 15366295

